# Construction of cost-effective bimetallic nanoparticles on titanium carbides as a superb catalyst for promoting hydrolysis of ammonia borane†

**DOI:** 10.1039/c7ra10568a

**Published:** 2018-01-03

**Authors:** Zhangwei Guo, Tong Liu, Qingtao Wang, Guanhui Gao

**Affiliations:** College of Ocean Science and Engineering, Shanghai Maritime University Haigang Ave 1550 201306 Shanghai China; College of Materials Science and Engineering, Qingdao University of Science and Technology Zhengzhou Rd 53 266000 Qingdao China liutong@qust.edu.cn; Paul-Drude-Institut für Festkörperelektronik Hausvogteiplatz 5–7 10117 Berlin Germany gao@pdi-berlin.de

## Abstract

Bimetallic cost-effective CoNi nanoparticles (NPs) are conveniently supported on titanium carbides (MXene) by a simple one-step wet-chemical method. The synthesized CoNi/MXene catalysts are characterized by XPS, TEM, STEM-HAADF and ICP-AES. The as-prepared CoNi NPs with a size of 2.8 nm are well dispersed on the MXene surface. It is found that among the CoNi bimetallic system, Co_0.7_Ni_0.3_ shows the best performance toward catalyzing ammonia borane (AB) decomposition with a turnover frequency value of 87.6 mol_H_2__ mol_cat_^−1^ min^−1^ at 50 °C. The remarkable catalytic performance is attributed to the mild affiliation of MXene to NPs, which not only stabilizes NPs to maintain a good dispersion but also leaves sufficient surface active sites to facilitate the catalytic reaction.

## Abstract 2

Bimetallic cost-effective CoNi nanoparticles are supported on MXene by a simple one-step wet-chemical method. The Co_0.7_Ni_0.3_/MXene shows the best performance toward catalyzing AB decomposition with TOF of 87.6 mol_H_2__ mol_cat_^−1^ min^−1^ at 50 °C.

## Graphical abstract

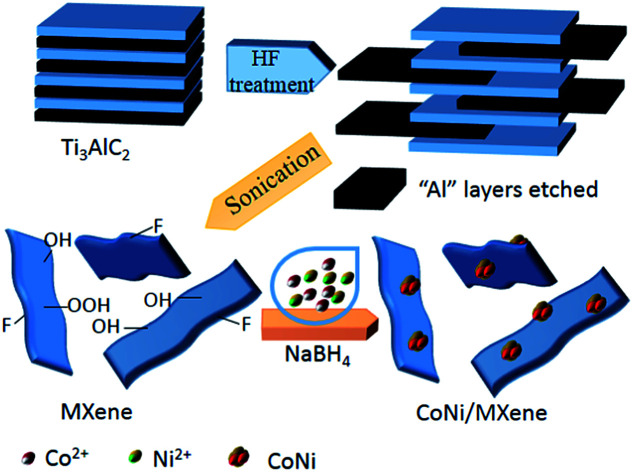

## Introduction

In order to solve the global problems caused by heavy use of fossil fuels in the world, it is highly desirable to develop clean and sustainable energy sources and in turn decrease the consumption of traditional fossil fuels.^1–5^ Hydrogen, as an environmentally clean energy carrier, has been regarded as one of the most promising candidates to meet the increasing demands for an efficient and clean energy supply.^6–9^ Among various chemical hydrogen storage materials, ammonia-borane (AB) with a high hydrogen content (19.6 wt%), high stability under ambient conditions, high solubility and nontoxicity, has been considered as a leading contender in promising chemical hydrogen-storage materials for various applications.^10–12^ Up to now, intensive efforts have been made to achieve efficient AB decomposition, generating 3 mol of H_2_ per mole of AB, through an efficient, economical and durable catalyst. Although, noble metal-based catalysts, such as Pt, Rh, and Ru,^13^ provided the highest activities to dehydrogenation of AB, the high cost and scarcity of these catalysts hinders their industrial application. Alternatively, cost-effective metals have been recently developed. Especially, it is found that bimetallic systems usually show higher catalytic kinetics than their monometallic counterparts, due to the synergistic geometric and electronic effects of bimetallic systems, such as Cu–Ni, Cu–Co, Co–Ni.^14–16^ However, the aggregation of these catalysts always resulted in the tremendous loss of their active sites. To address this problem, different carriers such as graphene, metal–organic frameworks and carbon nanotubes were used to disperse metal nanoparticles (NPs).^17–20^ The results show that these carriers can effectively restrain the agglomeration of metal NPs and improve their catalytic activities. Recently, graphene-like transition metal carbide (MXene, Ti_3_C_2_(OH_*x*_F_1−*x*_)_2_) has been widely investigated as a promising carrier for new nanocatalyst.^21–26^ The abundant functional groups on MXene surface, such as Ti–OH and Ti–F bonds, not only stabilize the nanoparticles during reduction but also improve the hydrophilicity of the resultant catalysts.^27,28^ However, there is rare report on catalytic properties of Ti_3_C_2_X_2_ supported NPs. In this work, for the first time, we synthesize a composite material of CoNi bimetallic nanoparticles supported on MXene *via* a simple one-step wet-chemical method. Unexpectedly, the resultant CoNi/MXene nanocatalysts with 100% of H_2_ selectivity show extremely high catalytic activity and excellent durability toward AB decomposition.

## Experiment

### Synthesis of CoNi/MXene

**Scheme 1:**
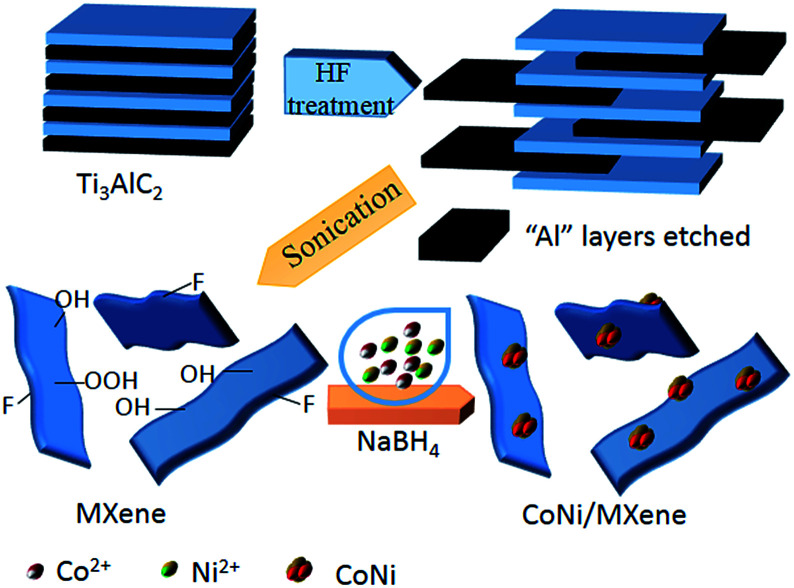
Schematic representation of synthesis of the CoNi/MXene catalyst.

The synthesis route of the CoNi/MXene nanocatalyst is shown in Scheme 1. In a typical experiment, 100 mg MXene was dissolved in 2 mL water in a two-neck round-bottom flask (30 mL). Ultrasonication was required to get a uniform dispersion for 30 min. Then, 200 μL cobalt chloride solution (0.07 mmol mL^−1^) and 200 μL nickel chloride (0.03 mmol mL^−1^) were added into the MXene solution. The resulted mixture was stirred for 20 min with a rotating shaker (220 rpm). A 0.5 mL aqueous solution of sodium borohydride (NaBH_4_) (1.3 mol L^−1^) was quickly added into the resulted mixture under vigorous stirring for 3 h at 0 °C using an ice bath to maintain the temperature. When the situ synthesis reaction was completed, 0.5 mL of aqueous solution containing 1 mmol AB was injected into the mixture using a syringe, a gas burette filled with water was connected to the reaction flask to measure the volume of released gas. For comparison, CoNi/vulcanxc-72 carbon (CoNi/XC-72), CoNi/graphene oxide (CoNi/GO) and CoNi/Al_2_O_3_ reduced by NaBH_4_ were synthesized respectively by the similar method.

### Morphological, structural and compositional characterizations

Transmission electron microscopy (TEM) images and high-resolution STEM measurements were obtained using Tecnai G2 F30 S-Twin instrument with a field emission gun operating at 200 kV. X-ray photoelectron spectroscopy (XPS) measurement was performed with ESCALAB 250Xi spectrophotometer. The metal contents of the catalyst were analyzed using inductively coupled plasma atomic emission spectroscopy (ICP-AES) on Leeman PROFILE SPEC. Mass spectrometry (MS) analysis of the generated gas was performed using an OmniStar GSD320 mass spectrometer, wherein Ar was chosen as the carrying gas.

## Results and discussions

**Fig. 1:**
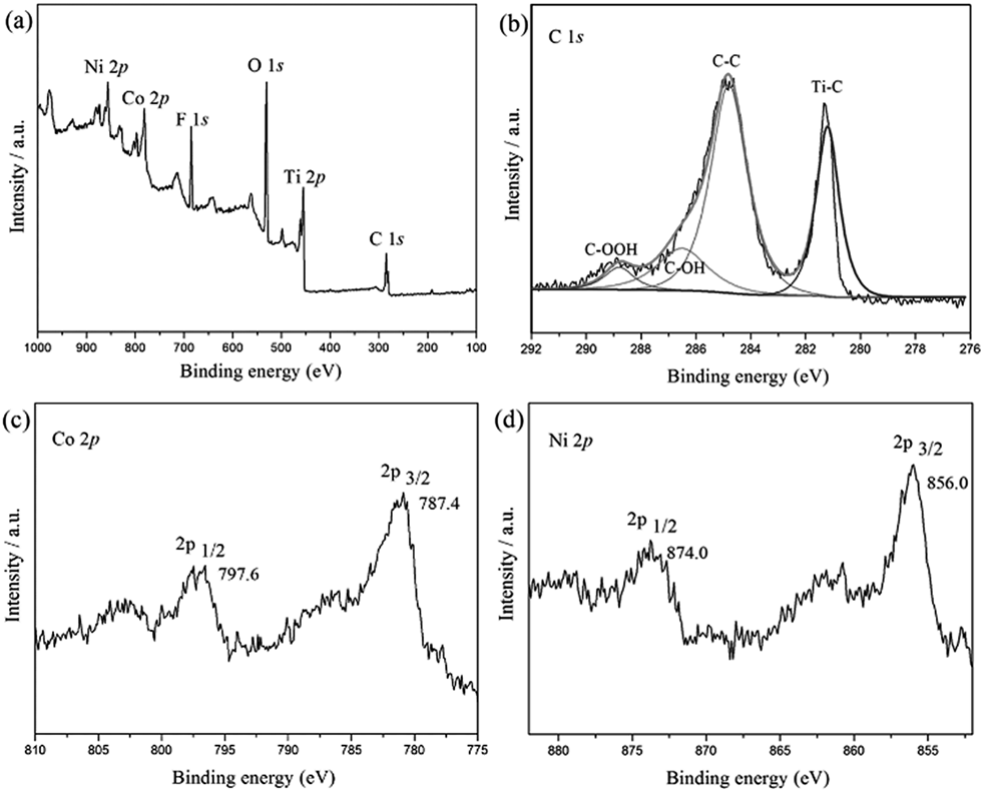
Spectroscopic characteristics of Co_0.7_Ni_0.3_/MXene. Survey spectrum of Co_0.7_Ni_0.3_/MXene (a), and XPS spectra of C 1s, Co 2p and Ni 2p (b–d).

Fig. 1 shows XPS spectra of CoNi/MXene in the survey, and C 1s, Co 2p and Ni 2p regions. As shown in Fig. 1a, the C 1s spectrum can be divided into several peaks, which corresponds to different kinds of bonds including C–Ti, C–C, C–OH, and HO–C=O, respectively. Additionally, the presence of F and O indicate the introduction of functional groups such as OH and F on Ti_3_C_2_X_2_ surface (Fig. S1 and S2†), which not only enhances the hydrophilicity of the support but also facilitates to the stabilization of the synthesized CoNi NPs, leading a fine dispersion and good stability of the synthesized nanocatalyst in this heterogeneous system. The two peaks located at 787.4 eV and 797.6 eV can be assigned to elemental Co^0^ 2p_3/2_ and Co^0^ 2p_1/2_, respectively. While the Ni peaks corresponding to Ni^0^ 2p_3/2_ and Ni^0^ 2p_1/2_ are observed at 856 eV and 874 eV, respectively.^27,28^ From the above results, it can be clearly demonstrated that the CoNi particles have been successfully anchored on MXene surface.

**Fig. 2:**
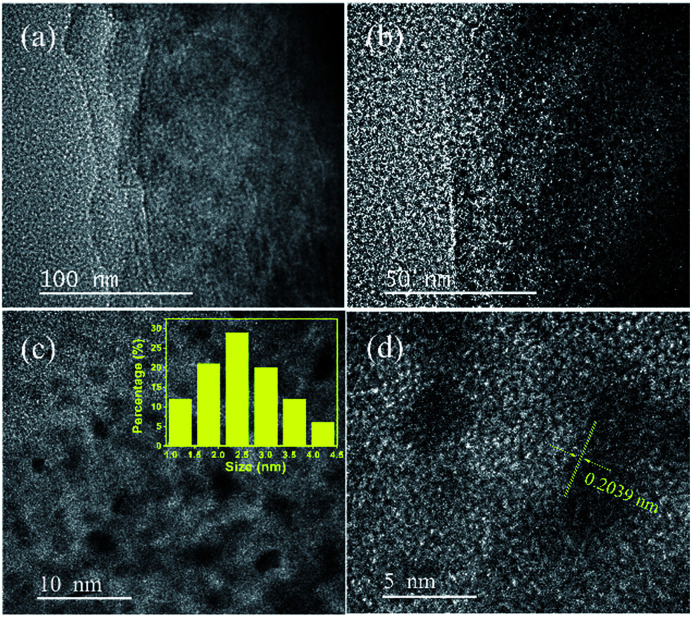
TEM images (a–c), and HRTEM images (d) of the Co_0.7_Ni_0.3_/MXene.

**Fig. 3:**
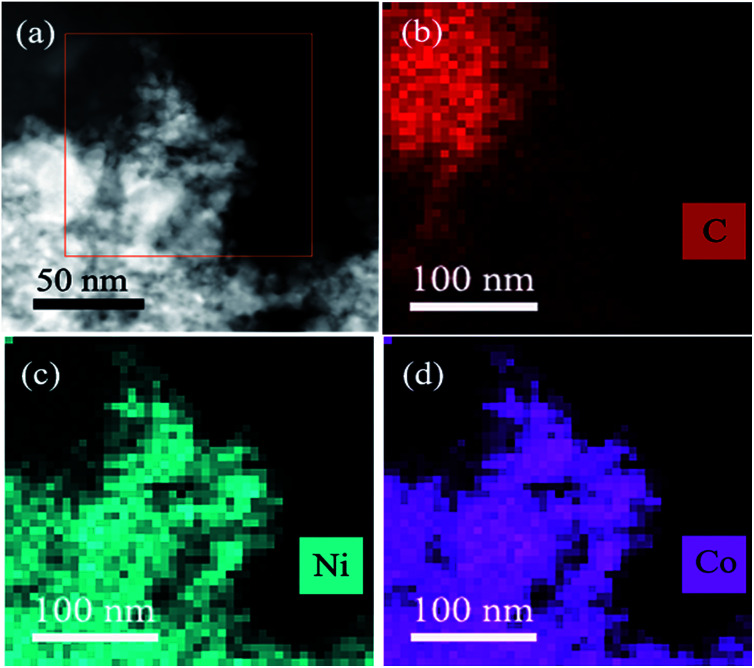
The STEM-HAADF images of the Co_0.7_Ni_0.3_/MXene (a) and the corresponding elemental mapping images for C (b), Ni (c) and Co (d).

To further investigate the morphologies of resultant CoNi NPs, transmission electron microscopy (TEM) and high resolution TEM (HRTEM) are used. As seen in Fig. 2a and S3,† the Co_0.7_Ni_0.3_ NPs have the good dispersion and no obvious aggregation has been happened. The average diameter of the NPs is about 2.8 nm (Fig. 2c inset). The narrow size and shape distributions benefit from the ‘confine effect’ from the functional OH and F groups on Ti_3_C_2_X_2_ surface, which has been confirmed by XPS results. A high resolution TEM (HRTEM) analysis of a representative nanoparticle of Co_0.7_Ni_0.3_ suggests that the as-synthesized specimen has a clearly identified lattice fringe space of 0.2039 nm, which is less than that of the (111) plane of pure face-centered cubic (fcc) Co (0.2046 nm), and larger than that of the (111) plane of pure face-centered cubic (fcc) Ni (0.2033 nm). These results confirmed the as-synthesized Co_0.7_Ni_0.3_ NPs with an alloy structure. In addition, an energy dispersive X-ray (EDX) spectrum proves the presence of Co and Ni elements with an atomic ratio of 2.35 : 1 (Fig. S4†), which is in good agreement with the ICP-AES results (Table S1†). The elemental mappings of Ni, Co, and C (Fig. 3) further reveal that Co and Ni elements were homogeneously dispersed throughout the whole MXene. From the above results, it can be seen that the as-synthesized CoNi nanocatalysts have been well dispersed on MXene surface, which benefits to enhancing catalytic property for AB decomposition in this bimetallic heterogeneous system.

**Fig. 4:**
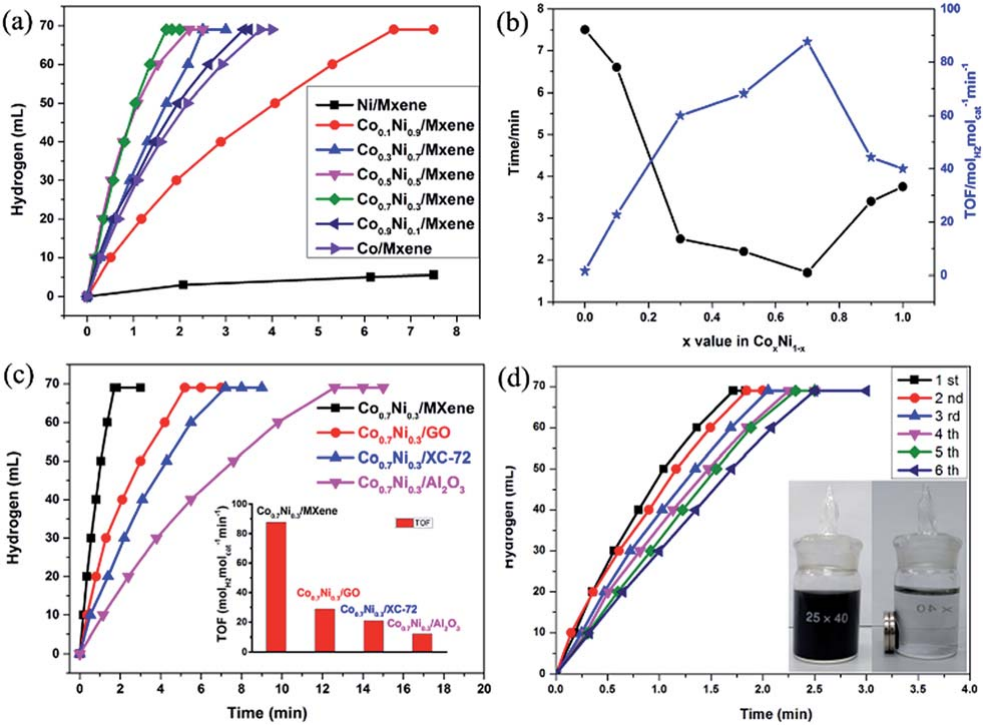
Time course plots for H_2_ generation from AB decomposition with different Co/Ni molar ratios (0 ≤ *x* ≤ 1) (a); the corresponding reaction time and TOF (b); over Co_0.7_Ni_0.3_/MXene, Co_0.7_Ni_0.3_/GO, Co_0.7_Ni_0.3_/XC-72 and Co_0.7_Ni_0.3_/Al_2_O_3_ at 50 °C (c); catalyzed by Co_0.7_Ni_0.3_/MXene from 1st to 6th cycles (d). The molar ratio of metal/AB = 0.02.

The as-synthesized Co_*x*_Ni_1−*x*_/MXene (0 ≤ *x* ≤ 1) NPs have been applied as catalysts for the AB decomposition at 50 °C with a constant molar ratio of catalyst/AB = 0.02 (Fig. 4a). A fast hydrogen generation starts immediately without any induction period when the catalyst is mixed with AB solution under magnetic stirring at 50 °C. Namely, Co_0.7_Ni_0.3_ NPs exhibit the highest catalytic activity for AB decomposition, and the corresponding TOF value can achieve 87.6 mol_H_2__ mol_cat_^−1^ min^−1^ at 50 °C. This value is one of the highest values among all the heterogeneous catalytic dehydrogenation of AB (Table 1). Mass spectrometry (MS) results further confirm the generated H_2_ and the absence of NH_3_ in the released gas (Fig. S5†). Notably, the composition of Co and Ni has the obvious effect on the catalytic performance of Co_*x*_Ni_1−*x*_ for AB decomposition. If absence of Co additives, reaction catalyzed by pure Ni shows the lowest catalytic activity and hydrogen generation quantity. By alloying Co to Ni, the catalytic activity and selectivity is significantly improved. When the molar ratio of Co (*x* value) increases from 0.1 to 0.7, the reaction time decreases from 6.6 min to 1.7 min and the corresponding TOF value increases from 22.7 mol_H_2__ mol_cat_^−1^ min^−1^ to 87.6 mol_H_2__ mol_cat_^−1^ min^−1^, respectively. However, when the molar ratio of Co reaches 1.0, namely, no addition of Ni, the reaction is completed in 3.75 min. Obviously, the catalytic activity of the Co_*x*_Ni_1−*x*_ alloy is better than those of pure Ni and Co. The reason for the enhanced performance of Co_*x*_Ni_1−*x*_ alloy may be mainly resulted from the synergetic effect between Co and Ni, which can efficiently tune surface electronic states of bimetallic nanoparticles, particularly related to local strain and effective atomic coordination number at the surface, leading an apparent improvement for AB decomposition.

**Table 1:** Catalytic activities of different catalysts for AB decomposition

Catalyst	Temp. (K)	TOF (mol_H_2__ mol_cat_^−1^ min^−1^)	*E*_a_ (kJ mol^−1^)	Ref.
CoNi/MXene	323	87.6	36.9	This work
	303	39.1	36.9	This work
CuCo@MIL-101	298	19.6	—	15
Cu_0.8_Co_0.2_O@graphene oxide	298	70	43.5	29
Cu@Co/Co	303	8.36	51.3	30
NiCu@C nanofiber	298	3.6	285.9	31
CuCo_2_O_4_/Ti	298	44	23.6	32
Cu_0.49_Co_0.51_/C	298	45	51.9	33
Ni/CNTs	298	26.2	32.3	16
Co/PSMA	298	25.7	34	34
Co/graphene	298	18.5	27.41	35
Co/rGO	298	13.8	32.7	36
Cu_0.1_@Co_0.45_Ni_0.45_/graphene	298	15.5	58	37
Au–Co@CN	298	28.4	—	38
Ag@Co/graphene	298	10.23	20.03	8
Au@AuCo/CNT	298	13.24	41.9	39
Pd@Co/graphene	298	408	—	40
Cu@FeNi	303	114	44	41
Cu@FeCo	303	10.5	38.75	42

Furthermore, the carrier materials also play an important role on determining the catalytic performance for AB decomposition. In order to evaluate the effect of the carrier materials on the catalytic performances toward the AB decomposition, Co_0.7_Ni_0.3_ NPs are loaded on different carriers such as GO (Co_0.7_Ni_0.3_/GO), XC-72 (Co_0.7_Ni_0.3_/XC-72), and Al_2_O_3_ (Co_0.7_Ni_0.3_/Al_2_O_3_) and their catalytic activities toward AB decomposition at 50 °C with a constant molar ratio of catalyst/AB = 0.02 are shown in Fig. 4c. The AB decomposition catalyzed by Co_0.7_Ni_0.3_/GO, Co_0.7_Ni_0.3_/XC-72 and Co_0.7_Ni_0.3_/Al_2_O_3_ are completed in 5.2, 7.2 and 12.6 min, respectively, and the corresponding TOF values are 28.8, 20.6 and 11.9 mol_H_2__ mol_cat_^−1^ min^−1^ in the same reaction condition. Compared to Co_0.7_Ni_0.3_/GO, Co_0.7_Ni_0.3_/XC-72 and Co_0.7_Ni_0.3_/Al_2_O_3_, the prepared Co_0.7_Ni_0.3_/MXene show higher catalytic kinetics. The superior catalytic performance is mainly attributed that strong interface interaction between metal and carrier, especially for the defect-rich or oxygen group-deficient surfaces on MXene. Such interface interaction is considered to favour the formation of a tunable electronic state of metal NPs, which enhances the AB decomposition.

In order to obtain the activation energy (*E*_a_) of the AB decomposition catalyzed by Co_0.7_Ni_0.3_/MXene catalysts, the reactions at different temperatures (30–60 °C) were carried out. Fig. S6a† shows that the plots of the generated H_2_*versus* reaction time for AB decomposition in the presence of Co_0.7_Ni_0.3_ catalyst at different temperatures. The Arrhenius plot of ln TOF *vs.* 1/*T* for the catalyst is plotted in Fig. S6b,† and *E*_a_ is calculated to be 36.9 kJ mol^−1^.

For the practical application of catalysts, the durability/stability of catalysts is the key point. Therefore, the durability of the Co_0.7_Ni_0.3_/MXene nanocatalyst up to fifth run for AB decomposition was characterized by adding additional aliquots (1 mmol) of AB to the catalyst after the reaction completion for the last run. It is evident from Fig. 4d, it is found that a little decrease after a six-time recycle test was seen and the reaction time is prolonged from 1.7 to 2.5 min, indicating the as-prepared Co_0.7_Ni_0.3_/MXene possess a moderated durability in AB decomposition. It is due to that the functional OH and F groups on Ti_3_C_2_X_2_ surface, as an anchor, can stabilize the CoNi NPs, avoid the aggregation of CoNi NPs during the reaction process, which is confirmed by TEM images. As clearly seen from the TEM images (Fig. S7†), the CoNi NPs can well disperse on MXene surface and there is no obvious aggregation of the CoNi NPs on MXene. Furthermore, the *in situ* synthesized NPs are magnetic and thus can be separated from the reaction solution by an external magnet (Fig. 4d, inside), which makes the practical recycling application of the NPs more convenient.

## Conclusions

In summary, cost-effective bimetallic CoNi NPs are well dispersed on MXene surfaces by a simple one-step wet-chemical method. Wherein the MXene plays an important role in stabilizing the CoNi NPs, leading a fine dispersion and good stability of the synthesized nanocatalyst in this heterogeneous system. By optimizing the fraction of Co component in Co–Ni system, the synthesized Co_0.7_Ni_0.3_/MXene have been proven to the most reactive nanocatalyst in this family reward to AB decomposition with 100% of H_2_ selectivity and excellent catalytic performance of 87.6 mol_H_2__ mol_cat_^−1^ min^−1^ at 50 °C. Additionally, the synergetic effect between CoNi NPs and MXene also improve the kinetic toward AB decomposition. This simple synthetic method can be easily extended to facile preparation other MXene supported metal NPs.

## Conflicts of interest

There are no conflicts to declare.

## Supplementary Material

RA-008-C7RA10568A-s001

## References

[cit1] Young S. (2001). Nature.

[cit2] Edwards P. P., Kuznetsov V. L., Brandon N. P. (2008). Energy Policy.

[cit3] Yang X., Xu Q. (2016). Chin. J. Catal..

[cit4] Wang H. L., Zhu Q. L., Zou R., Xu Q. (2017). Chem.

[cit5] Kotowicz J., Bartela L., Wecel D., Dubiel K. (2017). Energy.

[cit6] Chen H. M., Chen C. K., Liu R. S., Zhang L., Zhang J., Wilkinson D. P. (2012). Chem. Soc. Rev..

[cit7] Graetz J. (2009). Chem. Soc. Rev..

[cit8] Yang L., Luo W., Cheng G. (2013). ACS Appl. Mater. Interfaces.

[cit9] Cheng J., Gu X., Liu P., Zhang H., Ma L., Su H. (2017). Appl. Catal., B.

[cit10] Yan J. M., Wang Z. L., Wang H. L., Jiang Q. (2012). J. Mater. Chem..

[cit11] Metin O., Duman S., Dinc M., Ozkar S. (2011). J. Phys. Chem. C.

[cit12] Chandra M., Xu Q. (2006). J. Power Sources.

[cit13] Zhan W. W., Zhu Q. L., Xu Q. (2016). ACS Catal..

[cit14] Yu C., Fu J., Muzzio M., Shen T., Su D., Zhu J., Sun S. (2017). Chem. Mater..

[cit15] Li J., Zhu Q. L., Xu Q. (2015). Catal. Sci. Technol..

[cit16] Feng W., Yang L., Cao N., Du C., Dai H. M., Luo W., Cheng G. Z. (2014). Int. J. Hydrogen Energy.

[cit17] Zhu Q. L., Li J., Xu Q. (2013). J. Am. Chem. Soc..

[cit18] Song F. Z., Zhu Q. L., Yang X. C., Xu Q. (2016). ChemNanoMat.

[cit19] Chen W. Y., Ji J., Duan X. Z., Qian G., Li P., Zhou X. G., Chen D., Yuan W. K. (2014). Chem. Commun..

[cit20] Akbayrak S., Ozkar S. (2012). ACS Appl. Mater. Interfaces.

[cit21] Xie X., Chen S., Ding W., Nie Y., Wei Z. (2013). Chem. Commun..

[cit22] Naguib M., Come J., Dyatkin B., Presser V., Taberna P.-L., Simon P. (2012). Electrochem. Commun..

[cit23] Xie X., Xue Y., Li L., Chen S., Nie Y., Ding W., Wei Z. D. (2014). Nanoscale.

[cit24] Ghidiu M., Lukatskaya M. R., Zhao M. Q., Gogotsi Y., Barsoum M. W. (2014). Nature.

[cit25] Naguib M., Kurtoglu M., Presser V., Lu J., Niu J., Heon M., Hultman L., Gogotsi Y., Barsoum M. W. (2011). Adv. Mater..

[cit26] Peng Q., Guo J., Zhang Q., Xiang J., Liu B., Zhou A., Liu R., Tian Y. (2014). J. Am. Chem. Soc..

[cit27] Li X., Fan G., Zeng C. (2014). Int. J. Hydrogen Energy.

[cit28] Li X., Zeng C., Fan G. (2015). Int. J. Hydrogen Energy.

[cit29] Feng K., Zhong J., Zhao B., Zhang H., Xu L., Sun X., Lee S.-T. (2016). Angew. Chem., Int. Ed..

[cit30] Du Y. S., Cao N., Yang L., Luo W., Cheng G. Z. (2013). New J. Chem..

[cit31] Yousef A., Barakat N. A. M., El-Newehy M., Kim H. Y. (2012). Int. J. Hydrogen Energy.

[cit32] Liu Q. B., Zhang S. J., Liao J. Y., Feng K. J., Zheng Y. Y., Pollet B. G., Li H. (2017). J. Power Sources.

[cit33] Bulut A., Yurderi M., Ertas I. E., Celebi M., Kaya M., Zahmakiran M. (2016). Appl. Catal., B.

[cit34] Metin Ö., Özkar S. (2011). Int. J. Hydrogen Energy.

[cit35] Li M., Hu J., Lu H. (2016). Catal. Sci. Technol..

[cit36] Yang L., Cao N., Du C., Dai H., Hu K., Luo W., Cheng G. (2014). Mater. Lett..

[cit37] Meng X., Yang L., Cao N., Du C., Hu K., Su J., Luo W., Cheng G. (2014). ChemPlusChem.

[cit38] Guo L. T., Cai Y. Y., Ge J. M., Zhang Y. N., Gong L. H., Li X. H. (2014). ACS Catal..

[cit39] Yang L., Luo W., Cheng G. (2013). ACS Appl. Mater. Interfaces.

[cit40] Wang J., Qin Y. L., Liu X., Zhang X. B. (2012). J. Mater. Chem..

[cit41] Wang H. L., Yang J. M., Wang Z. L., Jiang Q. (2012). Int. J. Hydrogen Energy.

[cit42] Qiu F., Dai Y., Li L., Xu C., Huang Y., Chen C. (2014). Int. J. Hydrogen Energy.

