# Supplementary material for: Construction of cost-effective bimetallic nanoparticles on titanium carbides as a superb catalyst for promoting hydrolysis of ammonia borane
Source: RSC Adv. 2018 Jan 3;8(2):843–7. doi: 10.1039/c7ra10568a (PMC9077009; doi:10.1039/c7ra10568a)
Supplement: RA-008-C7RA10568A-s001 [file RA-008-C7RA10568A-s001.pdf]

Electronic Supplementary Material (ESI) for RSC Advances.
This journal is © The Royal Society of Chemistry 2017

Supplementary Information

Construction of cost-effective bimetallic nanoparticles on titanium carbides as a superb catalyst for promoting hydrolysis of ammonia borane

Zhangwei Guo,‡[a] Tong Liu,‡*[b] Qingtao Wang[b] and Guanhui Gao*[c]

[a] *College of Ocean Science and Engineering, Shanghai Maritime University, Haigang Ave 1550, 201306 Shanghai ,China.*

[b] *College of Materials Science and Engineering, Qingdao University of Science and Technology, Zhengzhou Rd 53, 266000 Qingdao, China.*

[c] *Paul-Drude-Institut für Festkörperelektronik, Hausvogteiplatz 5–7, 10117 Berlin, Germany.*

1. Materials

Ammonia borane (NH_3•BH_3, Aladdin reagent Co., Ltd, >97%), nickel(II) chloride hexahydrate ($NiCl_2·6H_2O$, Sinopharm Chemical Reagent Co., Ltd, >98%), cobalt(II) chloride hexahydrate ($CoCl_2·6H_2O$, Sinopharm Chemical Reagent Co., Ltd, 99%), sodium borohydride ($NaBH_4$, Sinopharm Chemical Reagent Co., Ltd, >96%), vulcanxc-72 carbon (Cabot corporation, ≥97 %), Al_2O_3 (Sigma-Aldrich Co., Ltd, 99%), were used without further purification. De-ionized water with the specific resistance of 18.2 MΩ·cm was obtained by reversed osmosis followed by ion-exchange and filtration.

2. Synthesis of Mxene:

The support MXene was initially prepared at room temperature using Ti_3AlC_2 as raw material and concentrated hydrofluoric acid as the etching agent. Specifically, concentrated hydrofluoric acid (40%, 100 mL) was slowly added to a beaker containing Ti_3AlC_2 (5 g). The solution was stirred for 24 h to remove the Al layer in Ti_3AlC_2. The black precipitate was then separated from the as-prepared suspension by centrifugation and washed several times with distilled water and absolute ethanol. Finally, the product was dried under vacuum at 60 C for 12 h.

3. Durability testing of the catalysts:

For testing the durability of CoNi/MXene catalysts, 1 mmol AB was subsequently added into the reaction flask after the completion of the first-run decomposition of AB.

Such test cycles of the catalyst for the decomposition of AB were carried out for 5 runs at 50 °C by adding AB.

4. Calculation of turnover frequency (TOF)

The TOF reported here is an apparent TOF value based on the number of Ni and Co atoms in catalyst, which is calculated from the equation as follow:

$$TOF= P_0V / (RTn_{NiCo}t)$$

Where P_0 is the atmospheric pressure (101325 Pa), V is the final generated volume of H_2 gas, R is the universal gas constant (8.3145 m^3 Pa mol^{-1} K^{-1}), T is the room temperature (298 K), n_{NiCo} is the total mole number of Ni and Co atoms in catalyst and *t* is the completion time of the reaction in minute.

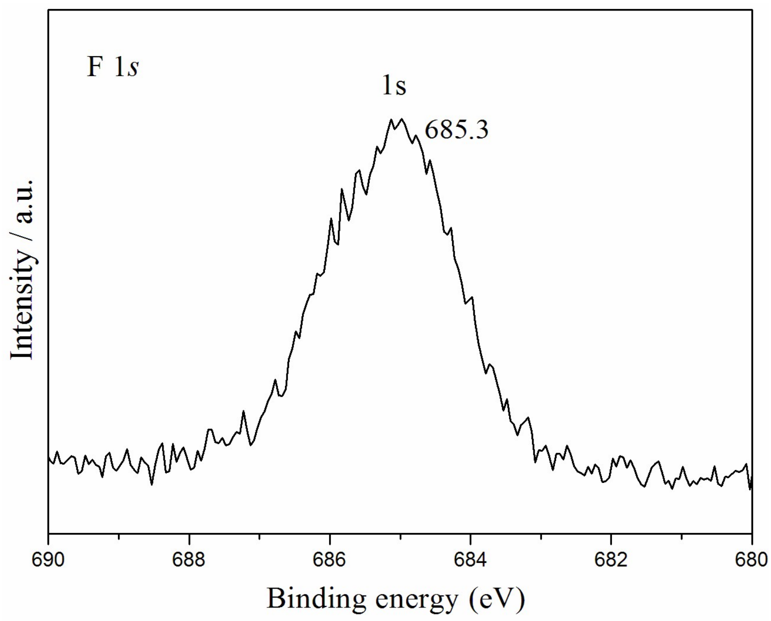

Fig. S1. XPS spectrum of F 1s for CoNi/MXene.

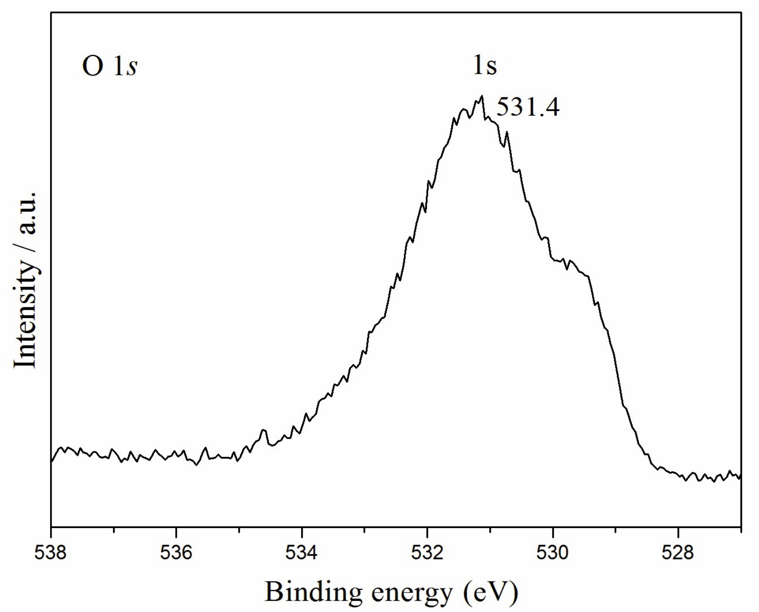

Fig. S2. XPS spectrum of O 1s for CoNi/MXene.

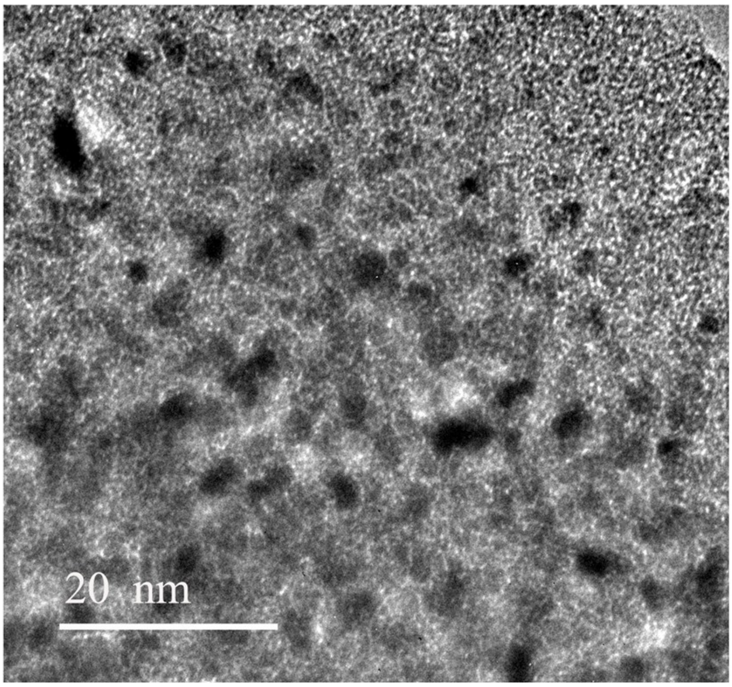

Fig. S3. TEM images of the $Co_{0.7}Ni_{0.3}$/MXene.

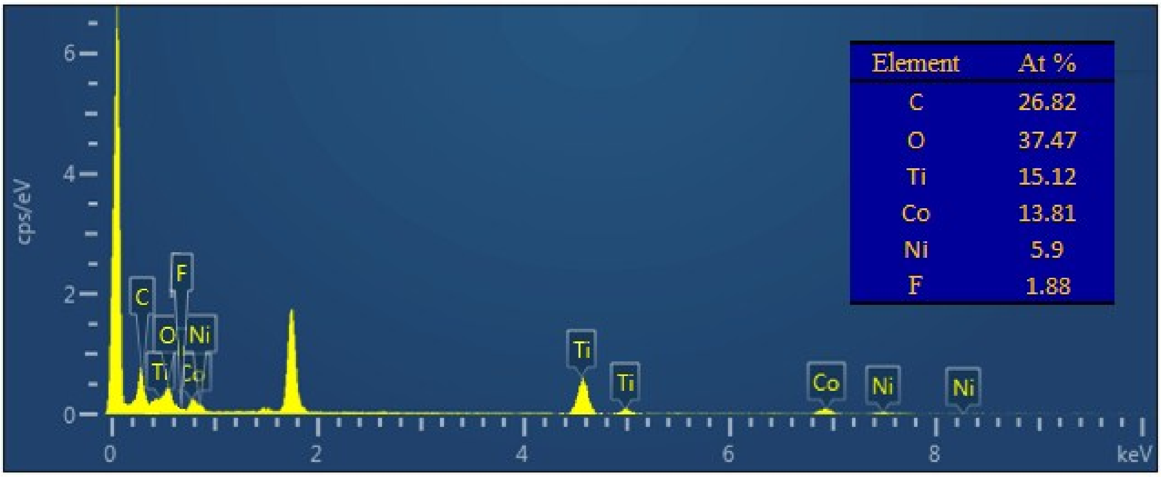

Fig. S4. EDX spectra for the prepared CoNi/MXene catalyst.

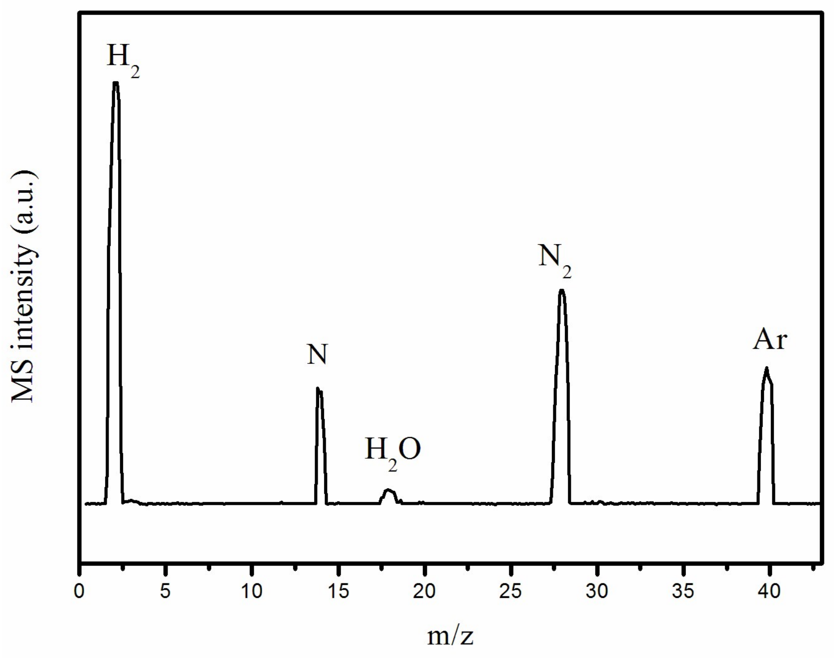

Fig. S5. Mass spectrometry profile for the gases released from the decomposition reaction of AB catalyzed by $Co_{0.7}Ni_{0.3}$/MXene at 50 ℃.

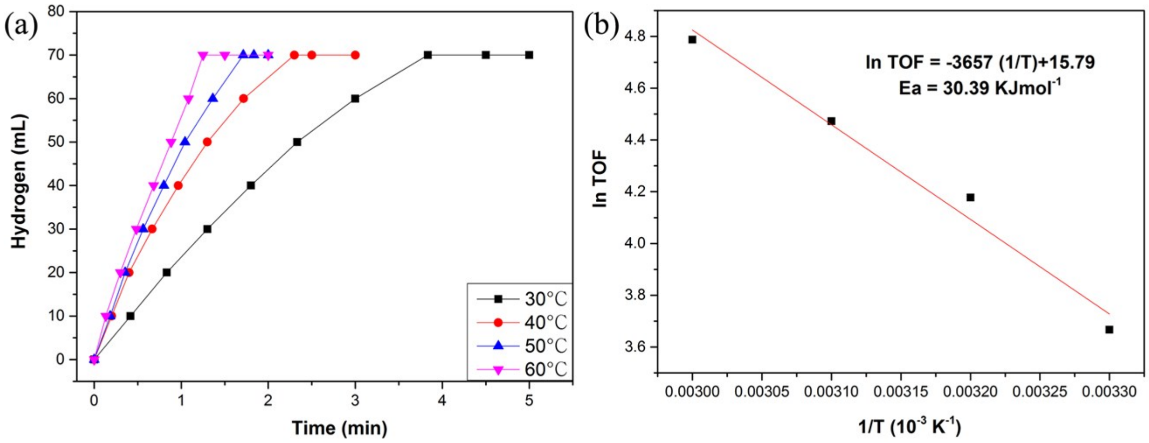

Fig. S6. Time course plots for H_2 generation from AB decomposition catalyzed by $Co_{0.7}Ni_{0.3}$/MXene at different temperatures (a); arrhenius plot (ln(TOF) vs. 1/T (b). The molar ratio of metal/AB = 0.02.

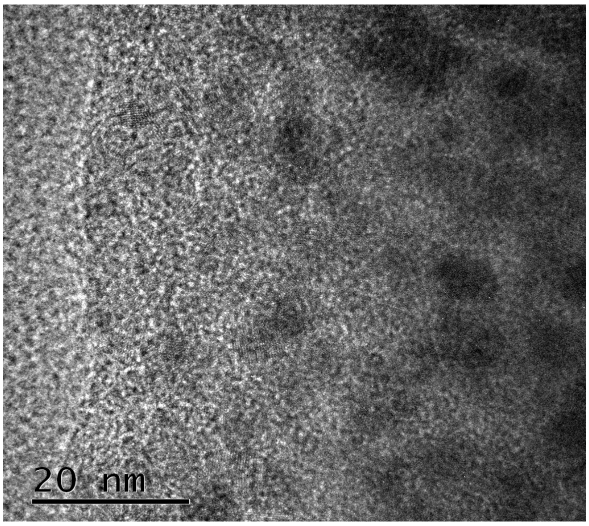

Fig. S7. TEM image of the $Co_{0.7}Ni_{0.3}$/MXene NPs after the sixth cycle.

Table S1 ICP-AES results of different catalysts

Catalyst	Co Ni finial molar ratio
Co/MXene	100:0
$Co_{0.9}Ni_{0.1}$/MXene	88:12
$Co_{0.7}Ni_{0.3}$/MXene	72:28
$Co_{0.5}Ni_{0.5}$/MXene	53:47
$Co_{0.3}Ni_{0.7}$/MXene	31:69
Ni/MXene	0:100
